# Effects of *Eucommia ulmoides* Leaf Extract on the Technological Quality, Protein Oxidation, and Lipid Oxidation of Cooked Pork Sausage During Refrigerated Storage

**DOI:** 10.3390/foods14030441

**Published:** 2025-01-29

**Authors:** Yanan Zhao, Wenhui Wang, Yuqi Wu, Qimeng Sun, Jinfeng Pan, Xiuping Dong, Shengjie Li

**Affiliations:** 1 School of Food Science and Technology, Dalian Polytechnic University, Dalian 116304, China; zyndlpu@163.com (Y.Z.); fumei1998@126.com (W.W.); wuyuqi0722wu@163.com (Y.W.); sqm18943033617@163.com (Q.S.); pjf613@163.com (J.P.); dxiuping@163.com (X.D.); 2 Collaborative Innovation Center for Marine Food Deep Processing, Dalian Polytechnic University, Dalian 116034, China; 3 National Engineering Research Center of Seafood, Dalian 116304, China

**Keywords:** cooked sausage, *Eucommia ulmoides* leaf extracts, technological quality, protein oxidation

## Abstract

The present research work was based on evaluating the effects of *Eucommia ulmoides* leaf extract (EULE) on the technological quality and protein oxidation of cooked pork sausage during refrigerated storage. Sausages were manufactured with different levels of EULE (0, 0.15, and 0.3 g/kg) and stored at 4 °C for 3, 20, and 40 d, respectively. Quality attributes including cooking loss, texture, and color were evaluated, and the total carbonyl and total sulfhydryl as well as the specific markers α-aminoadipic acid semialdehyde (AAS) and lysinonorleucine (LNL) were analyzed for protein oxidation. The results revealed that the inclusion of EULE exhibited effectiveness in reducing the formation of protein carbonyls, particularly AAS and LNL, while inhibiting the loss of total sulfhydryl. Nevertheless, EULE increased the cooking loss, hardness, and chewiness of the sausages compared to the control group. These findings demonstrated that EULE could be considered a potential natural antioxidant for use in sausage production.

## 1. Introduction

Meat and meat products are rich in proteins and lipids, both of which are susceptible to oxidative reactions during processing and storage, leading to quality deterioration of the products [1]. Numerous studies have focused on lipid oxidation in meat and meat products, including its consequences, mechanisms, and inhibition [2]. Moreover, as the main component of muscle tissue, protein plays a decisive role in affecting the organoleptic, nutritional, and technological aspects of meat products [3]. Though less noticeable than lipid oxidation and microbial spoilage, protein oxidation has been shown to play important roles impacting protein functionalities, which could jeopardize food quality. Protein oxidation undermines the structural integrity of protein molecules by inducing alterations in amino acid side chains, leading to the fragmentation of polypeptide chains and the establishment of intermolecular crosslinks within proteins [4]. These structural modifications play a crucial role in the degradation and modification of functional properties, such as protein gelation, water retention capacity, and digestibility [5]. However, these functional properties of proteins are significantly correlated with the organoleptic characteristics of meat products, including color, texture, and flavor [6]. Moreover, the oxidation of proteins alters the amino acid composition, resulting in the production of oxidative by-products, which include detrimental compounds such as carbonyl α-aminoadipic acid semialdehyde (AAS) and lysinonorleucine (LNL) [7]. Such modifications not only reduce the bioavailability and nutritional quality of proteins but may also be associated with a range of health issues, including hyperlipidemia, hypertension, and Alzheimer’s disease, thereby representing a considerable risk to human health [8]. Consequently, it is imperative to reduce protein oxidation throughout the processing and storage phases to improve the quality of sausages, extend their shelf life, and protect consumer health.

Cooked sausage is a popular meat product and is eaten daily worldwide. Different antioxidant strategies have been implemented in the meat industry to minimize the oxidative deterioration of protein and lipids, such as utilizing active packaging and incorporating antioxidant food additives [9]. An example of this is the use of 1.5% tomato (*Solanum lycopersicum* L.) extract, which modifies gelatin coatings to enhance antioxidant properties and prolong the shelf life of food products [9]. In particular, owing to the ongoing debates regarding the safety and toxicity of synthetic antioxidants, more attention has been paid to natural antioxidants derived from plant sources not only because they can increase the nutritional values of meat products but also because they can improve the oxidative stability of lipids and proteins [10].

*Eucommia ulmoides* bark has long been used in traditional Chinese medicine; however, its leaves, rich in polyphenols, have been rarely explored for their bioactive compounds that can scavenge reactive oxygen species (ROS) [11]. *E. ulmoides* leaf extract (EULE) and BHT have been reported to exhibit comparable abilities to reduce the levels of thiobarbituric acid reactive substances (TBARS) and to preserve the color of fresh pork patties during refrigerated storage [12]. Nevertheless, limited information is available regarding the utilization of EULE as an antioxidant in cooked sausages and its impacts on the technological quality and protein oxidation of the products.

Therefore, the objective of this research was to investigate the impact of EULE on the technological quality and protein oxidation of pork sausages stored at 4 °C for a period of 40 d.

## 2. Materials and Methods

### 2.1. Materials and Chemicals

*E. ulmoides* leaf extract was obtained from Xi’an Baoyifeng Biochemical Technology Co. (Xi’an, China). Derived from the leaves of the plant Eucommiaceae, the extract is white in color, and its main phytochemical constituent is chlorogenic acid, at 98%. Three commercial crossbred pigs (males, 70–80 kg carcass weight) were slaughtered at a local abattoir, and fresh hind leg lean meat and back fat (one side of each carcass) were obtained from the pigs within 24 h of death. Meat samples were transported to the laboratory at 0–4 °C on ice and categorized into groups of sausages through a process of random assignment. Bicinchoninic Acid Assay (BCA) protein concentration determination kit was sourced from Solabao Technology Company (Beijing, China). Additional reagents and additives were procured from Solabao Technology Company (Beijing, China).

### 2.2. Manufacture of Cooked Sausage

The formulations for the experimental treatments of cooked pork sausages at three different levels of EULE addition are shown in Table 1. The sausage employed for each experimental treatment group consisted of 2 kg of sausage mix. The sausages were divided into three treatment groups: 0 g, 0.15 g, and 0.3 g of EULE per kg of sausage patty (56% lean pork, 14% pork fatback, and 30% ice), while the salt and other ingredients were incorporated based on g/100 g of the sausage mince. Randomly assigned lean pork from the hind leg and pork fatback were minced into small pieces and ground separately using a meat grinder (BJRJ-82, EXPRO, Beijing, China). The minced meat was classified into three separate categories: a control group containing 0 g/kg of EULE, an antioxidant group containing 0.15 g/kg of EULE, and a second antioxidant group with 0.3 g/kg of EULE. Subsequently, each batch of minced meat was amalgamated with the additional ingredients and processed individually utilizing a chopper (BZBJ-20, EXPRO, Beijing, China). Ice was added to maintain the temperature of the minced meat within the range of 5 to 6 °C. The minced meat was then consistently filled into 22 mm cellulose casings and maintained at 4 °C for 12 h. After the refrigeration period, the meat was subjected to cooking in a combi oven (SCC WE 101, Rational AG, Claus Hackenberg, Germany) until an internal temperature of 70 °C was reached. Upon completion of the cooking process, the sausages underwent a cooling phase in running water for 30 min. The pH value of the cooked sausages after cooking was around 6.0. Subsequently, they were vacuum-sealed (MULTIVAC, C100, Sepp Haggenmuller GmbH & Co. KG, Wolfertschwenden, Germany) and subjected to post-pack pasteurization (PPP) in a water bath (YUHUA, DF-101S, Gongyi City, China) at 90 °C for a duration of 3 to 4 s. The vacuum-sealed sausages were then stored in aluminum foil to protect them from light and stored at 4 °C for periods of 3, 20, and 40 d, respectively. The pork sausages utilized in this research were manufactured in three batches, with each batch being prepared on a separate day, at the Food Processing Laboratory of Dalian Polytechnic University in China. Each batch comprised three treatment groups (0, 0.15, and 0.3 g/kg of EULE) across three distinct storage durations (3, 20, and 40 days), and at each sampling time, three separate sausage replicates were taken for different analysis.

### 2.3. Determination of Cooking Loss

Cooking losses were evaluated by measuring the weight difference of pork sausages before and after the cooking process [13]. The cooking loss (%) was calculated by determining the mass difference between the pre-cooking and post-cooking stages, expressed as a percentage of the initial weight of the sausage.

### 2.4. Determination of Textural Properties

The sausages were cut into cylindrical specimens with a height of 2 cm, and Texture Profile Analysis (TPA) was performed on a Stable Micro Systems Texture Analyser (TA-XTN plus, Stable Micro Systems, Godalming, UK). The hardness, chewiness, and springiness of the cooked sausages were assessed using the TPA mode of the mass spectrometer. A P50 probe was attached to the Texture Analyser, and the method parameters were adjusted to 2 mm/s for the pre-test phase, 1 mm/s for the test phase, and 1 mm/s for the post-test phase. The trigger force was set to 5 g, and the compression ratio was set to 30% [14].

### 2.5. Measurement of Color Stability

The cooked sausage was divided into cylindrical samples with a height of 2 cm and subsequently encased in plastic wrap to maintain a smooth cut surface free from imperfections such as holes and folds. The wrapped cut surface was then measured using a spectrophotometer (TS7700, 3nh, Guangdong SUNSHI Intelligent Technology Co., Shenzhen, China). The chosen measurement mode encompassed a diameter of 4 mm, and specular reflection was excluded from the analysis. After the correction process, two indices were obtained from the measurements: brightness (L*) and redness (a*). These indices collectively offer a thorough characterization of the color alterations observed in the cooked sausages [15].

### 2.6. Extraction of Total Protein

The cooked sausage was cut into small portions, after which 1 g of the sample was mixed with 25 mL of 5% sodium dodecyl sulfate (SDS). This mixture was then homogenized for 30 s at a speed of 8000 rpm. The homogenates (IKA, T 25 D S25, Staufen, Germany) were incubated in a water bath at 80 °C for a duration of 30 min, followed by centrifugation (TOMY, CAX-571, Nerima-ku, Tokyo 190-0073, Japan) at 3000 rpm for 20 min. After centrifugation, the supernatant was carefully collected and filtered through gauze. The concentration of protein in the resulting solution was then determined using a BCA Protein Quantification Kit (Solabao Technology Company, Beijing, China).

### 2.7. Determination of Total Carbonyl Content

An aliquot of 100 μL of the previously proposed total protein solution was taken at a concentration of 2 mg/m L. Subsequently, 1 mL of 10% trichloroacetic acid (TCA) was added to the aliquot, and the resulting mixture was centrifuged at 5000× *g* for 5 min at 4 °C. The precipitate was combined with 400 μL of 5% SDS and heated in a water bath at 100 °C for 10 min to facilitate dissolution. Dissolved samples were mixed with 0.8 mL of 0.3% (*w*/*v*) 2,4-dinitrophenylhydrazine (DNPH) solution (prepared with 3 M HCl), and blank samples were combined with only 0.8 mL of 3 M HCl. Following a 30-minute incubation in the dark at room temperature, 400 μL of 40% TCA was introduced, and the solution was centrifuged at 10,000× *g* for 5 min at 4 °C. The protein precipitates were then subjected to three washing cycles using 1 mL of a 1:1 (*v*/*v*) ethyl acetate–ethanol solution. Subsequently, the precipitates were resuspended in 1.5 mL of 6 M guanidine hydrochloride solution and 20 mM PBS (pH 6.5), incubated overnight at 4 °C, and centrifuged at 1000× *g* for 10 min prior to the final assay. The samples were finally analyzed for their carbonyl content (nmol of carbonyl/g of protein) at 280 (protein concentration) and 370 nm (carbonyls) [16].

### 2.8. Determination of Total Sulfhydryl Content

Total sulfhydryl content was determined by reacting 2 mg/mL total protein with DTNB solution according to the method studied by [17] with some modification. Briefly, 0.5 mL total protein samples (1.5 mg/mL) were mixed with 2 mL 100 mM Tris buffer (pH 8.0) and 0.5 mL DTNB reagent (10 mM DTNB in 100 mM Tris, pH 8.0) and incubated in the dark for 30 min at room temperature. Subsequently, the absorbance was measured at 412 nm using a UV–Vis Lambda 35 spectrophotometer (PerkinElmer, Waltham, MA, USA). The sample blank was prepared using 0.5 mL of 100 mM Tris buffer without DTNB, while the reagent blank was prepared with 0.5 mL of 5% SDS, 100 mM Tris buffer instead of the total protein sample. The sulfhydryl content was calculated using a molar extinction coefficient of 13,600 L/(mol·cm).

### 2.9. Sample Preparation for HPLC-MS/MS Analysis

The sample preparation technique utilized in this research was aligned with the methodology outlined by [18]. The total protein sample, obtained through Section 2.6 and exhibiting a concentration of 2 mg/mL, underwent precipitation with 1 mL of cold 10% TCA. Following this, the sample was centrifuged at 600× *g* for 10 min at 4 °C. The pellets were washed with 1.5 mL of cold 5% TCA and centrifuged at 1200× *g* for 5 min. Following this procedure, the pellets were reconstituted in 0.5 mL of 4-morpholineethanesulfonic acid (MES) buffer. Then the sample was derivatized with 0.5 mL of MES buffer, which included 50 mM p-aminobenzoic acid (ABA), in conjunction with 0.25 mL of 100 mM sodium cyanoborohydride (NaCNBH3) also dissolved in MES buffer. The derivatization reaction for the synthesis of AAS-ABA was performed at a temperature of 37 °C under dark conditions for 90 min. The reaction was terminated by adding 0.5 mL of cold 50% TCA, and the mixture was centrifuged at 5000× *g* for 5 min. The protein precipitates were then washed once with 1 mL of 10% TCA and twice with 1 mL of a 1:1 (*v*/*v*) mixture of ethanol and ether, and the nitrogen-dried protein precipitates were hydrolyzed with 6 M HCl at 110 °C for 18 h. Following hydrolysis, the hydrolysates were dried under a continuous flow of nitrogen at 60 °C for 500 μL. Subsequently, the samples were reconstituted with a specified volume of Milli-Q water and filtered in preparation for HPLC-ESI-MS analysis.

### 2.10. HPLC-MS/MS Analysis

AAS and LNL were determined using the method reported by [16]. The determination of AAS and LNL was carried out using an LC-20AD high-performance liquid chromatography (HPLC) system (Shimadzu Corp., Kyoto, Japan) coupled with a 4000QTRAP mass spectrometer (AB Sciex Pte. Ltd., Framingham, MA, USA). Samples of 2 μL were introduced into an LC-20AD high-performance liquid chromatography (HPLC) system, which was outfitted with a CORTECS C18 reversed-phase column (2.7 µm, 4.6 × 150 mm; Waters Corporation, Milford, MA, USA). Quantification was achieved through external standardization utilizing standard curves that encompassed concentrations ranging from 10 nM to 100 nM of AAS-ABA, while the linearity for LNL was determined in the range of 2 to 100 ng/mL. AAS-ABA was prepared by us, and LNL was purchased from Santa Cruz Biotechnology (Dallas, TX, USA). The mass-to-charge ratios (*m*/*z*) for the precursor ion and the product were identified as 267 and 249 (AAS-ABA)/276 and 84 (LNL), respectively.

### 2.11. Determination of TBARS Content

The methodology utilized for the evaluation of TBARS levels was derived from previous studies, with slight adjustments made to enhance its application [19]. In the preparation of the sample, 1 g of cooked sausage mince was accurately weighed, followed by the addition of 0.5 mL of a 0.2% butylated hydroxytoluene (BHT) solution and 5 mL of 10% TCA. Following the centrifugation of the mixture at 10,000× *g* for 5 min at 4 °C, 1 mL of the supernatant was extracted and combined with 1 mL of 0.02 mM thiobarbituric acid (TBA) solution while a standard curve was established using a 25 μM 2,2,5,5-tetramethylpiperidine (TMP) solution. The centrifuge tubes containing the mixture were subjected to incubation in a water bath at 85 °C for 45 min. Following this incubation, the mixture was permitted to cool to room temperature and was centrifuged at 1000× *g* for 10 min at 4 °C, after which the supernatant was removed for testing. The absorbance of the resulting supernatant was then quantified at a wavelength of 532 nm utilizing an enzyme marker (Infinite M200, TECAN, Männedorf, Switzerland).

### 2.12. Statistical Analysis

In this study, we prepared three separate batches of sausages, of which each batch contained three treatments (0, 0.15, and 0.3 g/kg EULE) in triplicate (*n* = 3), and they were analyzed after 3, 20, and 40 days of storage at 4 °C. The findings of this study were derived from three replications, with the data for each group presented as means accompanied by standard error (S.E.) values. A general linear model (GLM) was employed to conduct an analysis of variance (ANOVA), where storage time and treatment were treated as fixed factors along with the interaction of these effects, with replicates (batches) being a random term. The statistical analysis was performed using R (version 4.2.1, R Foundation for Statistical Computing, Vienna, Austria). Significant differences between means at the 5% level (*p* < 0.05) were assessed through Duncan’s multiple range test.

## 3. Results and Discussion

### 3.1. Technological Quality

#### 3.1.1. Cooking Loss

**Figure 1:**
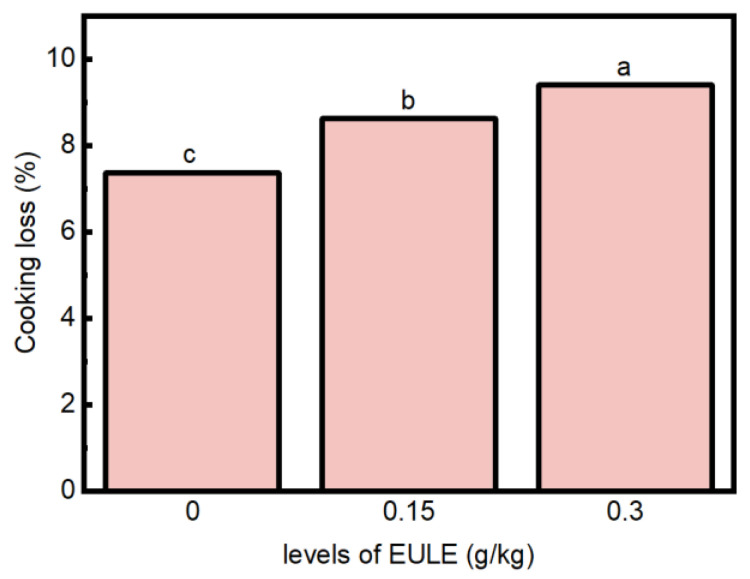
Effect of the levels of EULE on the cooking loss of cooked sausage. Values represent the mean of three replicates ± standard error (S.E.). Different letters indicate significant differences (*p* < 0.05).

Cooking loss has been considered a crucial parameter that reflects the ability of cooked sausages to retain water during the cooking process [20]. The cooking loss of meat products is affected by the binding capacity of their primary constituents, such as protein and fat [21]. Figure 1 illustrates that incorporating EULE significantly increased the cooking loss of cooked sausages (*p* < 0.05), and the more EULE was added, the higher the cooking loss was. Certain natural polyphenolic compounds, such as cyanidin 3-O-glucoside, cyanidin 3-O-rutinoside, caffeic acid, quercetin, and rutin, have been reported to interact with proteins, which can subsequently alter the structural and functional properties of proteins [22]. Similarly, ref. [23] reported that the introduction of chlorogenic acid disrupted the gel matrix of myofibrillar protein obtained from Coregonus Peled, leading to higher cooking loss. The water loss during cooking may be attributed to the denaturation of meat proteins during the cooking process, which leads to structural alterations such as the disruption of cell membranes, contraction of muscle fibers, and gelation of myofibrillar and sarcoplasmic proteins [24]. The capacity of proteins to engage with water molecules and retain them within the gel network structure is referred to as the water-holding capacity of proteins, which is predominantly influenced by the secondary and tertiary structural features of the proteins [25,26]. Therefore, the elevated cooking loss in the sausages with EULE could be ascribed to modifications in the protein structure induced by the abduction of the phenolic compounds.

#### 3.1.2. Textural Properties

**Figure 2:**
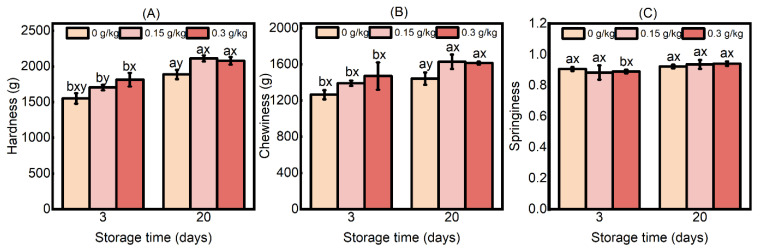
Effects of the levels of EULE on the texture of cooked sausage during refrigerated storage. (**A**): Hardness; (**B**): Chewiness; (**C**): Springiness. Values represent the mean of three replicates ± standard error (S.E.). The *p*-values of the interaction effects of EULE treatment and storage time on hardness, chewiness, and springiness, respectively, were 0.189, 0.391, and 1.030. Different letters (a,b) indicate significant differences (*p* < 0.05) among groups subjected to varying storage durations, and different letters (x,y) denote significant differences (*p* < 0.05) between groups that were stored for the same duration but contained varying amounts of EULE.

The textural properties of cooked sausages play a crucial role in shaping consumer preferences and acceptance, which are intrinsically associated with the overall sensory experience of the consumer [27]. The hardness of the sausage was independently affected by both EULE treatment and storage time (Figure 2A; *p* < 0.05). The hardness of the sausages within each group demonstrated a gradual increase that was associated with the length of the storage period. The hardness of the sausages in the EULE group was determined to be higher than that of the control group, but no statistically significant difference was detected between the hardness of the sausages in the 0.15 g/kg EULE group and the 0.3 g/kg EULE group (*p* > 0.05). The chewiness of the sausages was also independently impacted by EULE treatment and storage time (Figure 2B; *p* < 0.05). The changes in chewiness noted across the various treatment groups displayed a pattern similar to that of hardness. The analysis revealed no significant differences in the springiness of the sausages across varying storage durations (Figure 2C; *p* < 0.05). Furthermore, the springiness of the sausages in the group supplemented with EULE did not show a significant difference compared to the control group (*p* > 0.05).

Hardness is defined as the capacity of a sausage to withstand deformation, whereas chewiness pertains to the sensory experience associated with the act of chewing; hardness serves as a significant factor influencing chewiness, with a positive correlation frequently being observed between these two characteristics [28,29]. The observed increases in the hardness and chewiness of the cooked sausages can be attributed to the reduction in moisture content that occurred with prolonged storage time. This phenomenon aligns with the findings of a direct correlation between the moisture content of the sausage and its hardness [30,31]. On the other hand, numerous researchers have identified the occurrence of intramolecular and intermolecular cross-linking of oxidized proteins as a significant factor influencing the texture of meat products [17,32,33,34,35]. Furthermore, most studies have suggested that the formation of disulfide bonds in proteins serves as the primary mechanism for cross-linking in muscle food products [36]. In addition, α-aminoadipic acid semialdehyde (AAS), a characteristic carbonyl compound generated through the oxidation of proteins, has been demonstrated to be produced in significant quantities during the storage and processing of meat products [37]. Due to high reactivity, AAS can interact with the amino groups of lysine residues in adjacent proteins, leading to the formation of a non-disulfide-bond cross-linking agent referred to as lysinonorleucine (LNL) [18,38]. Recent research conducted by [37] indicates that protein carbonylation via AAS formation and protein cross-linking through the LNL formation seems to significantly contribute to the quality deterioration of high-oxygen modified atmosphere-packed beef. Furthermore, a review of the interaction between plant polyphenols and proteins suggests that under oxidative conditions, plant polyphenols may undergo oxidation to form quinones, which subsequently covalently bind to nucleophilic protein groups, which may also contribute to the promotion of additional protein cross-linking [39]. It has been proposed that the binding of polyphenols with proteins influences their functional qualities, including water-binding capacity, textural properties, proteolysis, thermal stability, and emulsification [40,41]. This phenomenon may elucidate that the observed increases in the hardness and chewiness of sausages in the EULE-treated group were higher than those in the control group at both 3 and 20 days of storage.

#### 3.1.3. Instrumental Color

**Figure 3:**
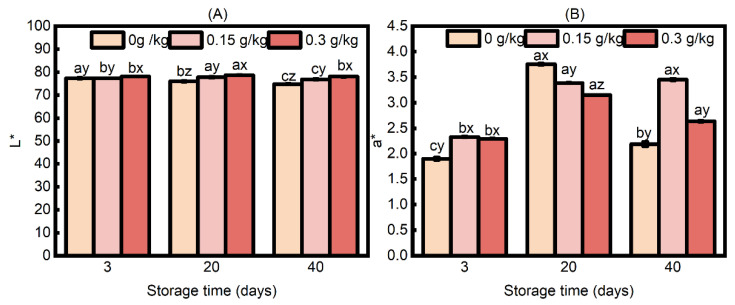
Effects of the levels of EULE on the color of cooked sausage during refrigerated storage. (**A**): L*; (**B**): a*. Values represent the mean of three replicates ± standard error (S.E.). The *p*-values of the interaction effects of EULE treatment and storage time on L* and a* were <0.001 and 0.012, respectively. Different letters (a–c) indicate significant differences (*p* < 0.05) among groups subjected to varying storage durations, and different letters (x–z) denote significant differences (*p* < 0.05) between groups that were stored for the same duration but contained varying amounts of EULE.

At the point of consumption, consumers usually consider the color of cooked meat to be one of the most important quality attributes [42]. The impact of EULE on the color of sausage during storage is illustrated in Figure 3. As depicted in Figure 3A, there exists an interaction effect between EULE treatment and storage time on the L* value of sausage color (*p* < 0.05). Following a three-day storage period, the L* values of sausages from the 0.3 g/kg EULE group exhibited a statistically significant increase compared to those from the 0 g/kg and 0.15 g/kg EULE groups (*p* < 0.05). On day 20, the L* values of the sausages in each group that received supplementation with EULE were higher than those in the control group. Additionally, a positive correlation was identified between the concentration of EULE administered and the observed L* values. On the 40th day of storage, the groups of sausages exhibited effects that were consistent with those observed on the 20th day. The L* value of the sausage in the 0 g/kg EULE group exhibited a gradual decline with the extension of storage time. Conversely, the incorporation of EULE increased the L* value of the sausages. This observed increase may be linked to the abovementioned finding that the inclusion of EULE led to an increase in cooking loss in sausages, which indicated diminished ability to retain water within the protein matrix. In general, as a result of diminished water retention, some of the loosely bound water is displaced from the three-dimensional network structure to the surface of the sausage, leading to an enhancement in brightness [43].

The variation in the a* value (redness) of sausage is primarily influenced by the processes of fat oxidation, protein oxidation, and myoglobin oxidation and is closely associated with the consumer’s sensory perception and acceptance of the products [12]. An interaction between EULE treatment and storage time was discovered for a* values (Figure 3B; *p* < 0.05). The a* values of the sausages in the 0 g/kg EULE group demonstrated a significant increase during the initial storage phase, which was subsequently followed by a marked decrease in the later storage period as time advanced (*p* < 0.05). At day 3, sausages subjected to treatment with EULE demonstrated elevated a* values relative to those in the control group Nevertheless, no significant difference was observed in the a* values between the sausages in the 0.15 g/kg and 0.3 g/kg EULE treatment groups. At day 20, the a* values of the sausages in the group supplemented with EULE were lower than those observed in the control group, and as the concentration of EULE increased, the a* values of the sausages decreased correspondingly. At day 40, the a* values of the control sausages exhibited a significant decrease compared to those observed at 20 days, whereas the a* values of the sausages in the 0.15 g/kg and 0.3 g/kg EULE groups were higher than the control treatment; moreover, the highest a* values were recorded in the 0.15 g/kg EULE group. During the sausage-making process, sodium nitrite (NaNO_2_) generates nitric oxide (NO) and facilitates the coordination of myoglobin (MbFe^2+^), resulting in the formation of a stable rose-red complex, nitrosomyoglobin, or Mb (Fe^2+^) NO [44]. This complex demonstrates enhanced stability relative to Mb (Fe^2+^) and exhibits a reduced vulnerability to oxidation. The thermal denaturation of nitrosomyoglobin leads to the production of bright red, heat-stable, non-browning nitrosylhemochrome [45]. This process may provide insight into the consistent enhancement of redness observed in sausages during storage durations of 3 to 20 days. However, a remarkable reduction in the a* value was observed after 20 days of storage in the control group, whereas for the 0.15 g/kg and 0.3 g/kg EULE groups, the sausages showed similar a* values between day 20 and day 40, indicating that the addition of EULE could prevent the fading of redness of sausages in the present study. As the storage duration of sausages extends, nitrite experiences both auto-degradation and reductive degradation, leading to a decrease in the synthesis of nitrosomyoglobin [46]. Furthermore, the storage process promotes the oxidation of lipids and proteins via a complex mechanism, which results in the production of large amounts of free radicals. These free radicals, in turn, expedite the oxidation of nitrosomyoglobin, ultimately contributing to a diminished redness in cooked sausages [44]. However, the phenolic compounds in EULE may also scavenge free radicals, potentially contributing to the stabilization of the red color in cooked pork sausage by delaying the oxidation of Mb (Fe^2+^) NO. In agreement with our study, ref. [47] demonstrated that rosemary oleoresin effectively preserved the redness of minced chicken packaged in a highly oxygenated environment.

### 3.2. Protein Oxidation

Protein oxidation in meat and meat products can be initiated through two primary mechanisms: direct initiation by reactive oxygen species (ROS) and reactive nitrogen species (RNS) or indirect initiation via secondary products resulting from oxidation reactions. Among these mechanisms, metal-catalyzed oxidative reactions and lipid oxidation are the most prevalent [48]. In the case of metal-catalyzed protein oxidation, reduced metal ions bind to specific sites on proteins and lead to the formation of highly reactive hydroxyl radicals (·OH) that subsequently target amino acids. Conversely, lipid oxidation may also be indirectly instigated by protein oxidation, occurring through interactions with lipid radicals or secondary products generated from lipid oxidation processes [49,50]. In this study, the levels of protein oxidation were measured by the determination of protein carbonyls and total thiol.

#### 3.2.1. Protein Carbonylation

**Figure 4:**
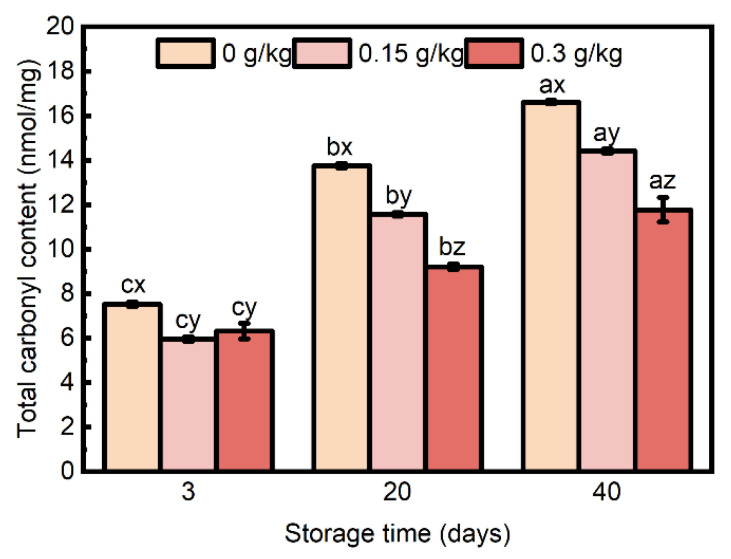
Effect of the levels of EULE on total carbonyls in cooked sausage protein during storage. Values represent the mean of three replicates ± standard error (S.E.). The *p*-value of the interaction of EULE treatment and storage time on total carbonyls was <0.001. Different letters (a–c) indicate significant differences (*p* < 0.05) among groups subjected to varying storage durations, and different letters (x–z) denote significant differences (*p* < 0.05) between groups that were stored for the same duration but contained varying amounts of EULE.

The formation of protein carbonyls has been commonly considered a marker for protein oxidation, and the 2,4-dinitrophenylhydrazine (DNPH) method has been commonly employed to quantify the total protein carbonyls [51]. There was an interaction effect of EULE treatment and storage time (Figure 4; *p* < 0.05) on the total carbonyl content. During storage, the total carbonyl content in the control group gradually increased and was significantly higher than that of the other treatment groups (*p* < 0.05). At 3 days, the total carbonyl content of the sausages in the 0.15 g/kg and 0.3 g/kg groups did not differ significantly and was lower than that of the control group. After 20 days of storage, the sausages’ total carbonyl content decreased with the incorporation of higher levels of EULE, indicating a dose-dependent effect. At day 40, the groups exhibited effects consistent with those observed after 20 days of storage.

**Figure 5:**
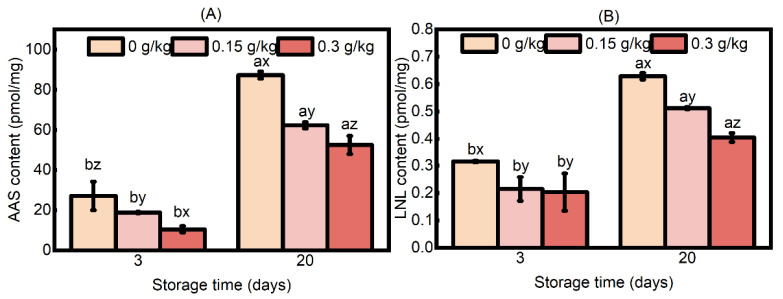
Effects of the levels of EULE on the AAS and LNL of cooked sausage protein during storage. (**A**): AAS; (**B**): LNL. Values represent the mean of three replicates ± standard error (S.E.). The *p*-values of the interaction effect of EULE treatment and storage time on AAS and LNL were <0.001 and 0.010, respectively. Different letters (a,b) indicate significant differences (*p* < 0.05) among groups subjected to varying storage durations, and different letters (x–z) denote significant differences (*p* < 0.05) between groups that have been stored for the same duration but have received varying amounts of EULE.

Though the DNPH method has been extensively used to determine the total carbonyl content, a notable drawback of this technique is its insufficient selectivity in investigating the mechanisms of protein carbonylation, which impedes a comprehensive understanding of the pathways associated with protein carbonylation [7]. Protein carbonyls are generated in multiple ways during meat processing, mainly through the attack of some amino acid residues by free radicals or the Maillard reaction, which results in deamination [52]. α-Aminoadipic semialdehyde (AAS) is a characteristic protein carbonyl formed through the protein carbonyl generation pathway described above, while lysinonorleucine (LNL) is a significant carbonyl derivative that arises from the further chemical reaction between AAS and adjacent lysine residues [18]. The quantitative analysis of characteristic protein carbonyls and their derivatives can yield valuable insights into the specific pathways of protein carbonylation, which, in turn, can help to analyze the intricate relationship between protein carbonylation and the deterioration of meat quality during processing and storage. Therefore, these two compounds may not only serve as biomarkers for protein oxidation but also directly contribute to the structural modification of proteins, subsequently affecting their functional properties [53]. The results showed an interaction effect of EULE treatment and storage time on AAS content (Figure 5A; *p* < 0.05). At both 3 and 20 days of storage, the levels of AAS in the sausages exhibited a gradual increase across all experimental groups (*p* < 0.05). Importantly, the AAS levels in the sausages supplemented with EULE were lower (*p* < 0.05) than those in the control group. Additionally, a negative correlation was observed between the concentration of EULE and the AAS content, indicating that higher concentrations of EULE were associated with reduced AAS levels (*p* < 0.05). The statistical analysis revealed an interaction between EULE treatment and storage time on LNL content (Figure 5B; *p* < 0.05). The LNL content of the sausages increased gradually in all treatment groups during the storage period. Notably, the LNL content of the sausage group treated with EULE was significantly lower than that of the control group (*p* < 0.05). Furthermore, an inverse correlation was observed between the LNL content and the amount of incorporated EULE on the 20th day of storage. The aforementioned results align with the impact of EULE on the total carbonyl content, indicating that EULE could effectively delay the onset of protein carbonylation in cooked sausage. Chlorogenic acid, specific iridoid glycosides such as eucommia glycosides, and genistein glycosides have been identified as the primary bioactive components of the leaves of *Eucommia ulmoides*, among which chlorogenic acid polyphenols are particularly noteworthy due to their significant antioxidant properties [54,55]. Phenolic compounds generally exhibit their protective effects via three primary mechanisms: hydrogen atom transfer, electron transfer–proton transfer, and sequential proton loss–electron transfer; chlorogenic acid primarily exerts its protective effects through the first pathway, which is associated with the bond dissociation energy (BDE) of the phenolic O-H bond. Chlorogenic acid possesses a low BDE, enabling it to effectively donate hydrogen to free radicals, thereby diminishing their reactivity [56]. This characteristic may contribute to a reduction in free radical activity within sausage systems, thereby offering protection to proteins against oxidation. Consequently, the presence of chlorogenic acid may mitigate the oxidative degradation of proteins in these systems. Furthermore, polyphenols interact with the amino groups of sausage proteins during high-temperature cooking, resulting in the formation of aminophenol adducts, and the interaction is particularly pronounced with the especially reactive amino acid lysine [39]. The attachment of polyphenols to the amine groups of lysine may impede carbonylation by stabilizing the structural integrity of lysine. These interactions may clarify the observed efficacy of the incorporation of EULE in reducing the overall carbonylation levels, as well as AAS and LNL, in cooked sausages.

#### 3.2.2. Total Sulfhydryl Content

**Figure 6:**
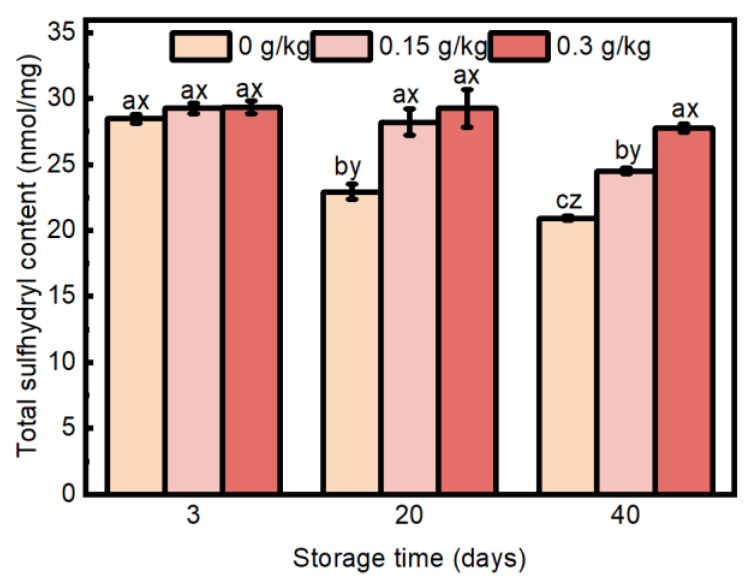
Effect of the levels of EULE on total sulfhydryl of cooked sausage protein during storage. Values represent the mean of three replicates ± standard error (S.E.). The *p*-value of the interaction effect of EULE treatment and storage time on total sulfhydryl was <0.001. Different letters (a–c) indicate significant differences (*p* < 0.05) among groups subjected to varying storage durations, and different letters (x–z) denote significant differences (*p* < 0.05) between groups that were stored for the same duration but contained varying amounts of EULE.

Sulfur-containing amino acids in proteins exhibit high sensitivity to oxidation, which can lead to the formation of various sulfur-containing compounds, including sulfonic acid and sulfinic acid, or result in the formation of protein cross-links through disulfide bonds; accordingly, the total sulfhydryl content is frequently employed as an indicator of the extent of protein oxidation [57]. An interaction effect of EULE treatment and storage time was observed on total sulfhydryl content (Figure 6; *p* < 0.05). The total sulfhydryl content in the control sausages exhibited a gradual decline over extended storage periods. No significant differential effect of EULE addition on the total sulfhydryl content of sausages was observed at 3 days of storage (*p* > 0.05). At 20 days, the total sulfhydryl content in the sausages from the group supplemented with EULE was significantly higher (*p* < 0.05) than that of the control group. Furthermore, no significant difference in total sulfhydryl content was found between the two EULE-supplemented groups (*p* > 0.05). On day 40, the total sulfhydryl content exhibited an increase corresponding to higher concentrations of added EULE, indicating that EULE has a dose-dependent influence on the total sulfhydryl content in the sausages. This observation indicates that EULE effectively prevents decreased total sulfhydryl content in cooked sausages. In agreement with this, ref. [58] showed that chicken sausages containing microencapsulated procyanidins displayed increased sulfhydryl content and exhibited enhanced antioxidant capacity relative to the other groups over a storage duration of 28 days at 4 °C. Ref. [59] incorporated essential oils derived from oregano, rosemary, and garlic into pork patties, demonstrating that the essential oils of rosemary and oregano significantly reduced the loss of protein sulfhydryl groups and inhibited the oxidation of sulfur-containing amino acids in proteins. In contrast, in two studies by refs. [60,61], both the incorporation of green tea extract into Bologna sausage and the addition of phenolic-rich white grape extract to raw beef patties packaged under high-oxygen conditions led to an increase in sulfhydryl loss, which were ascribed to the covalent addition reaction between the sulfhydryl groups of proteins and phenolic compounds, resulting in the formation of sulfhydryl–quinone adducts. In addition, the antioxidant properties are affected by the differing dosages of the same substance when administered in various additive amounts, as ref. [62] demonstrated that low concentrations of rosemary effectively inhibited the depletion of sulfhydryl groups in proteins, whereas high concentrations facilitated the covalent binding of polyphenols to these groups, leading to the formation of mercaptoquinone adducts. Ref. [63] noted that the amine group of lysine and the sulfhydryl group of cysteine display covalent binding to oxidized chlorogenic acid, exhibiting significantly higher reactivity relative to other amino acid side chains. The findings suggest that EULE exerts an inhibitory effect on the reduction in sulfhydryl content, likely due to its capacity to diminish free radical levels. This reduction in free radicals subsequently alleviates oxidative stress, thereby protecting sulfhydryl groups from potential damage. Additionally, polyphenols such as chlorogenic acid, present in EULE, may participate in a higher number of binding interactions with lysine amine groups in sausages. This interaction could contribute to the preservation of sulfhydryl groups, which is consistent with the observed inhibition of the increase in lysine-derived carbonylated substances, specifically AAS and LNL, by EULE.

### 3.3. Lipid Oxidation

**Figure 7:**
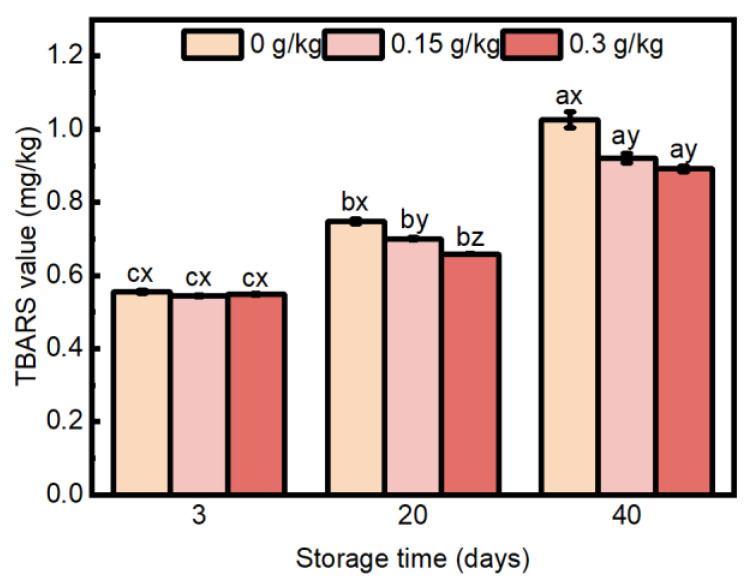
Effect of the levels of EULE on TBARS in the cooked sausages during storage. Values represent the mean of three replicates ± standard error (S.E.). The *p*-value of the interaction effect of EULE treatment and storage time on TBARS was <0.001. Different letters (a–c) indicate significant differences (*p* < 0.05) among groups subjected to varying storage durations, and different letters (x–z) denote significant differences (*p* < 0.05) between groups that were stored for the same duration but contained varying amounts of EULE.

Meat products are susceptible to lipid oxidation due to the large amount of polyunsaturated fatty acids in meat, and the thiobarbituric acid reactive substances (TBARS) value is a key indicator of lipid oxidation [64]. The results showed an interaction between EULE treatment and storage time for lipid oxidation (Figure 7; *p* < 0.05). TBARS values gradually increased in all treatments with longer storage time. After three days of storage, no significant differences (*p* > 0.05) were seen in the TBARS values of the sausages in the 0.15 g/kg and 0.3 g/kg EULE groups when compared to the control sausages. However, at 20 days of storage, the TBARS values of sausages containing EULE were significantly reduced (*p* < 0.05) compared to the control group. Furthermore, a significant decrease in TBARS values (*p* < 0.05) was detected with increasing levels of EULE. After 40 days of storage, the results were similar to those at 20 days. Still, no statistically significant difference was detected in the TBARS values of the sausages between the addition of 0.15 g/kg EULE and 0.3 g/kg EULE (*p* > 0.05). In a similar study, ref. [65] demonstrated that the addition of sea buckthorn extract, particularly the Vitaminnaja variety, at concentrations of 3 mg/kg and 5 mg/kg markedly reduced the production of malondialdehyde (MDA) in pork sausages. In a previous study, ref. [66] discovered that the inhibitory effect of *Eucommia ulmoides* extract on membrane lipid peroxidation across various lipid peroxidation models was associated with its polyphenolic composition. In 2010, ref. [12] established that the composition of EULE is characterized by a significant presence of phenolic compounds, including phenolic acids and flavonoids, also pointing out that this composition may contribute to its pronounced antioxidant properties. Phenolic antioxidants are thought to disrupt the oxidation process caused by free radicals by seizing hydrogen from the phenolic hydroxyl group, resulting in the formation of stable end products that hinder the onset or progression of lipid oxidation [67].

## 4. Conclusions

The present study demonstrated that *Eucommia ulmoides* leaf extract (EULE) had a significant antioxidant effect, especially inhibiting the generation of AAS and LNL. Nevertheless, the concomitant rise in cooking loss and changes in textural characteristics suggest that the addition of EULE could adversely impact the technological quality of the sausages. Given the significant antioxidant potential of EULE, it is imperative to conduct more comprehensive studies to clarify the underlying mechanism through which EULE jeopardizes the texture of cooked sausages and to find effective ways to control this negative effect.

## Figures and Tables

**Table 1:** Formulations of cooked pork sausages at three different levels of EULE addition.

Main Ingredients (%)			
Lean pork	56	56	56
Pork fatback	14	14	14
Ice	30	30	30
EULE (g/kg)	0	0.15	0.3
Salt and other ingredients (g/100 g)			
Sodium chloride	3	3	3
Sodium phosphate	0.4	0.4	0.4
Sodium lactate	3	3	3
Sodium nitrite	0.01	0.01	0.01
Sodium isoascorbate	0.1	0.1	0.1
Dextrose	1	1	1

## Data Availability

The original contributions presented in this study are included in the article. Further inquiries can be directed to the corresponding author.
